# Coculture of allogenic DBM and BMSCs in the knee joint cavity of rabbits for cartilage tissue engineering

**DOI:** 10.1042/BSR20170804

**Published:** 2017-11-09

**Authors:** Bin Xu, Rui Wang, Hao Wang, Hong-Gang Xu

**Affiliations:** 1 Department of Sport Injury and Arthroscopy, the First Affiliated Hospital of Anhui Medical University, Hefei 230022, P.R. China; 2 Department of Orthopedics, the Fourth Affiliated Hospital of Anhui Medical University, Hefei 230000, P.R. China

**Keywords:** Bone marrow derived mesenchymal stem cells, Cartilage tissue engineering, Decalcified bone matrix, Knee joint cavity, Rabbit

## Abstract

The present study aims to assess coculture of allogenic decalcified bone matrix (DBM) and bone marrow mesenchymal stem cells (BMSCs) in the knee joint cavity of rabbits for cartilage tissue engineering. Rabbits were assigned to an *in vitro* group, an *in vivo* group, and a blank control group. At the 4th, 8th, and 12th week, samples from all groups were collected for hematoxylin–eosin (HE) staining and streptavidin–peroxidase (SP) method. The morphological analysis software was used to calculate the average absorbance value (*A* value). SP and flow cytometry demonstrated that BMSCs were induced into chondrocytes. DBM scaffold showed honeycomb-shaped porous and three-dimensional structure, while the surface pores are interlinked with the deep pores. At the 4th week, in the blank control group, DBM scaffold structure was clear, and cells analogous to chondrocytes were scattered in the interior of DBM scaffolds. At the 8^th^ week, in the *in vivo* group, there were a large amount of cells, mainly mature chondrocytes, and the DBM scaffolds were partially absorbed. At the 12th week, in the *in vitro* group, the interior of scaffolds was filled up with chondrocytes with partial fibrosis, but arranged in disorder. In the *in vivo* group, the chondrocytes completely infiltrated into the interior of scaffolds and were arranged in certain stress direction. The *in vivo* group showed higher *A* value than the *in vitro* and blank control groups at each time point. Allogenic DBM combined BMSCs in the knee joint cavity of rabbits could provide better tissue-engineered cartilage than that cultivated *in vitro*.

## Introduction

Normal human articular cartilage is a relatively simple tissue without blood vessels or lymph, and it acquires the nutrients via the infiltration of synovial fluid, which in turn limits the proliferation of chondrocytes, therefore it has limited repair capabilities during a cartilage injury [1, 2]. The progressive loss of articular cartilage usually leads to the occurrence of osteoarthritis (OA), whose frequency is as high as 85% in the elderly people, which can even cause disability in later stages and seriously affects the quality of life in elder people [3, 4]. Luckily, cartilage tissue engineering offers a promising approach to repair cartilage injury via combining cells and biocompatible scaffold, so it is therapeutically possible to replace injured articular cartilage by tissue-engineered cartilage successfully [5]. However, no tissue-engineered cartilage can recapitulate the physicochemical properties of the autogenous bone especially the compression ability, and additionally, cartilage tissue engineering further focuses on the regeneration potential of immature *in vivo* engineered cartilage [6].

As an important therapeutic option for osteogenesis induction, decalcified bone matrix (DBM) has osteoconductive and osteoinductive properties that prompt bone regeneration, which approves it as a medical device in bone defects with a long record of clinical use in diverse forms [7]. With a certain elasticity and toughness, DBM could recover its original state automatically after compression [8]. Moreover, due to similar three-dimensional (3D) structure to autogenous bone, DBM possesses great biocompatibility mainly based on type I collagen (good scaffold for cell adhesion and growth) and high seed cell adhesion rate, which meets the requirements for cartilage tissue engineering on scaffold [9]. Bone marrow-derived mesenchymal stem cells (BMSCs), belonging to nonhematopoietic stem cells, were first reported by Feriedenstein in the early 1970s, and later, they were named as BMSCs in 1991 by Caplan as bone marrow possesses high potential of self-renewal and differentiation [10]. The application of stem cells, especially BMSCs, has achieved great success in cartilage and bone repair, and it facilitates better remodeling and integration with the host surface zone [11]. A previous study suggested that BMSCs are ideal seeding cells for bone tissue engineering, and the DBM loaded with BMSCs could be used as a good scaffold [12]. However, no research so far focused on their combined effects in knee joint cavity. Therefore, the present study aims to evaluate cartilage tissue engineering with allogenic DBM and BMSCs in the knee joint cavity of the rabbit, by comparing *in vitro* and *in vivo* cell–scaffold composites and *in vivo* DBM scaffolds.

## Material and methods

### Ethics statement

Experiments on animals were conducted in strict accordance with the approved animal protocols and guidelines established by Medicine Ethics Review Committee of the First Affiliated Hospital of Anhui Medical University. All efforts were made to minimize the suffering and pain of the animals.

### Experimental animals

Adult New Zealand white rabbits (3 months aged) were selected for the present study. They brought from the Lab Animal Center of Anhui Medical University. Dulbecco’s modified Eagle’s medium (DMEM) and fetal bovine serum (FBS) were purchased from Hyclone Inc. (Utah, U.S.A).; recombinant human transforming growth factor (TGF)-β1 and insulin-like growth factor (IGF)-1 were bought from Pepro Tech Inc. (New Jersey, U.S.A.); biotin goat anti-rabbit type II collagen was obtained from Bioss Biological Technology Company (Beijing, China). Immunohistochemistry staining kit (SP-9000) was bought from Zhongshan Biotechnology Company (Beijing, China). Carbon dioxide (CO_2_) incubator and inverted phase contrast microscope CKX41 were obtained from Olympus Corporation (Tokyo, Japan) and fluorescence microscope Eclipse 80i and camera control system from Nikon Corporation (Tokyo, Japan).

### Preparation of allogeneic DBM

Adult New Zealand white rabbits were used to obtain the limb extremities and iliac bones. All bones were decalcified in 0.5 mol/l hydrochloric acid (HCI) for 72 h (HCI:bone = 50 ml : 1 g), fat tissue was removed in absolute ether, the bone was dried, and later divided into pieces of dimensions of 3 mm × 3 mm × 3 mm, among which the relatively complete bone pieces (*n*=200) were separated into small containers and disinfected using ethylene oxide. Then, those small containers were randomly selected thrice for bacterial culture, and all the selected containers had no bacterial growth, which were further kept in the refrigerator at –20°C.

### Isolation, cultivation of BMSCs, and induction of BMSCs to chondrocytes

Bone marrow was extracted from 3-month-old male New Zealand white rabbits to prepare cell suspension by adding DMEM and erythrocyte lysis buffer. After counting, the cell suspension was inoculated in 2 × 10^4^/ml and cultivated in 5% CO_2_ at a constant temperature of 37°C for incubation and for cell passage till the third generation using adherent screening method. The 3rd generated BMSCs were stimulated to transform into chondrocytes by adding chondrocytes induction solution consisting of TGF-β1 (10 μg/l), IGF-1 (20 μg/l), and vitamin C (50 μg/l). Seven days after the stimulation of BMSCs, the slides of BMSCs and noninduced cells were observed for morphological changes and compared by type II collagen immunohistochemistry, namely, SP method [13]. The induced cells were adjusted into cell suspension of 2 × 10^6^/ml and were used as the seed cells in this experiment.

### Identification of BMSCs in rabbit

The immunohistochemistry was performed in the 3rd generated BMSCs to label CD105^+^ cells, which were analyzed by flow cytometer. Along with CD105^+^ antibody labeled by fluorescein isothiocyanate (FITC) as a marker and phosphate-buffered saline (PBS) solution as a control group instead of primary antibody, BMSCs were incubated at 4°C overnight and heated again at 37°C for an hour, followed by incubation in biotin goat anti-rabbit immunoglobulin (IgG) at 37°C for 30 min. Subsequently, BMSCs were incubated in SP reagents at 37°C for 30 min. At last, flow cytometer was performed to analyze the results.

### Construction and *in vitro* culture of cell–scaffold composite

A total of 30 disinfected DBM scaffolds were selected randomly and submerged in the seed cell suspension and stored into a 16-well plate. Based on a porous structure, the bone pieces could absorb the cell suspension and form cell–scaffold composites by 1-day culture, which was later cultivated *in vitro* under standard cell culture environment (37°C, 5% CO_2_ constant temperature incubation); fresh cell culture medium was replaced every 2–3 days) and was considered as the *in vitro* group.

### Establishment of rabbit models of osteochondral defect

A total of 20 New Zealand white rabbits weighed 2.2–2.5 kg were purchased from the Experimental Animal center of the Second Military Medical University (Shanghai, China). All the experimental animals were fed and managed by specially assigned person, and the operation in the whole experiment was performed strictly according to the Instructive notions with respect to caring for laboratory animals [14]. Each rabbit was anaesthetized using Su-Mian-Xin (0.2 ml kg^−1^) by intramuscular injection, then fixed on anatomy plate in supine position. After shed, their bilateral knee joint were sterilized using iodophor and ethyl alcohol. Their skins, subcutaneous, and joint capsule were cut apart layer by layer after creating an incision along the medial aspect of the knee joints, and then their distal femoral was totally exposed. In the center of trochlea, the cylindrical full-thickness defects of articular cartilage with a diameter of 4.0 mm were drill-down using trephinement, and then the blood was staunched by using sterile gauze.

### Cultivation of cell–scaffold composites and DBM scaffolds in the knee cavity of a rabbit

A total of 15 cell-scaffold composites were packed in muscle fascia(other materials) and sealed with several centimeters of sutures left at both the ends as markers. Then, they were implanted into the interstitial space of articular cartilage defects in the left knee of 15 adult male New Zealand white rabbites with suture knot fixed under the skin. Next, the joint capsule and skin were sutured in separate layers. It was considered as the in-vivo group.Similarly, 15 DBM scaffolds were implanted into interstitial space of cartilage defects in the right knee cavity of white rabbits as the blank control group. After surgery, the rabbits were fed in cages where they had the freedom to move. Intramuscular injection of penicillin was given for 3 days in order to prevent infection in the incision and the joint cavity. In the 4th, 8th, and 12th week after the surgery, rabbits were executed in order to get specimens for performing general observation, HE staining, and SP method. Eight different observations were randomly selected from immunohistochemical sections in the *in vitro* and the *in vivo* groups, followed by photographs using fluorescence microscope and camera control system (×200). Later, photographs were input into the Jetta 801D morphological analysis software to calculate the average absorbance value (*A* value).

### HE staining

After stripping out the attached soft tissues from samples, they were fixed with 4% paraformaldehyde at room temperature for 1 week and continuously decalcified with 10% EDTA for 4 weeks. The solution was changed every week. After that, excessive tissue was scraped away and the defect area and peripheral cartilage were retained. Routine fixation, dehydration, and paraffin embedding were done and 5 μm paraffin sections were taken. Subsequently, the sections were immersed in hematoxylin staining solution for 5 min after conventional dewaxing to water. After the floating color was washed away by running water, they were differentiated in 1% hydrochloric alcohol for seconds and washed by running water for 15 min. Then the sections were stained using HE for 2 min, separated the color by 95% alcohol for 1 min, dehydrated by gradient ethanol, and cleared by xylene. Finally, the sections were sealed by neutral balsam and observed under a microscope. Meanwhile, the bone shape of decalcification at the 12th week was observed using a scanning electron microscope (SEM).

### SP method

According to the method of Chen et al. [13], the cells were extracted from dewaxed paraffin sections and crawled on the slide. They were incubated with 3% hydrogen peroxide deionized-water at room temperature for 5–10 min to eliminate the activity of endogenous peroxidase. After that, they were washed with PBS and added with goat anti-rabbit type II collagen primary antibody labeled by biotin, and then incubated at 37°C for 15 min and washed with PBS again. Subsequently, after streptavidin solution labeled by horseradish peroxidase was added, the cells were incubated at 37°C for 15 min, washed with PBS, developed with DAB, and washed with running water. Then, they were stained using hematoxylin for 5 min, blued by distilled water for 15 min, dehydrated by gradient ethanol, and cleared by xylene. The cell slides were mounted with neutral balsam and observed under a microscope.

### Statistical analysis

Statistical analysis was performed using SPSS 21.0 (SPSS Inc., Chicago, IL, U.S.A.) software. Data calculated were expressed as mean ± standard deviation (SD). Completely random design was carried out to conduct variance analysis. Comparisons between groups were studied by *q*-test and repeated measures analysis of variance (ANOVA). *P*<0.05 was considered significantly different.

## Results

### Identification of rabbit BMSCs and induction of BMSCs to chondrocytes

**Figure 1:**
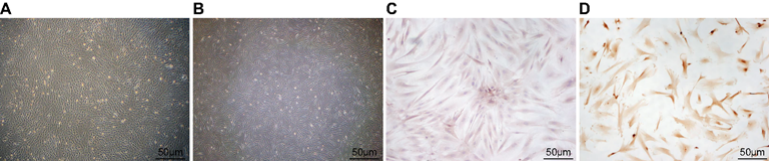
Identification of BMSCs and induction of BMSCs to chondrocytes (**A**) The 3rd generation of BMSCs for implantation, when the primary cells were inoculated for 13 days; (**B**) Morphology of the 3rd generation of BMSCs induced to chondrocytes (×100); (**C**) Type II collagen immunohistochemistry of the 3rd generation cells using SP method (×100); (**D**) Type II collagen immunohistochemistry after BMSC induction using SP method (×100); BMSCs, bone marrow-derived mesenchymal stem cells; SP, streptavidin–peroxidase.

The original generation of rabbit BMSCs were adherent to the glass wall after 48-h culture, it was grown in colonies for 4–5 days with noticeably increasing cells, and formed plenty of cell clones in different sizes. At that time, the fresh medium was replaced for the first time in 7 days, followed by cell passage every 10–14 days. The 3rd generation of BMSCs was observed under the microscope at high magnification: cells were long fusiform- or spindle-shaped with obvious nucleus, clear nucleolus, and high nucleus/cytoplasm ratio; vacuoles and lipid droplets were found in cytoplasm; and cells were closely adherent. After 48-h they were stimulated by adding chondrocytes induction medium, BMSCs changed their morphology from spindle-shaped to typical polygon-shaped chondrocytes, which were small in size and gradually merged into a single layer shown as “paving stone” (Figure 1A and B). SP method results are shown in Figure 1C and D. Figure 1C revealed that BMSCs at 3rd generation had a single nucleolus or several nucleoli in the nucleus without significant yellow staining in cytoplasm. After induction, the cell morphology turned into polygon, reniform, and fusiform. Figure 1D demonstrated that cells appearing as amount of brownish yellow particles in the cytoplasm and stroma, and their expression of type II collagen were positive.

### Expression of CD105+ in BMSCs

**Figure 2:**
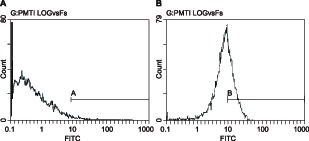
Expression of CD105^+^ in BMSCs by flow cytometer (**A**) The control group; (**B**) the 3rd generation of BMSCs; BMSCs, bone marrow-derived mesenchymal stem cells.

According to the results of flow cytometer (Figure 2), CD105^+^ cells accounted for 2.6% in the control group, while CD105^+^ cells accounted for 64.1% in the BMSCs at 3rd generation. Here, CD105^+^ was the surface marker of undifferentiated BMSCs.

### Repair of joint defect in the *in vivo* group

**Figure 3:**
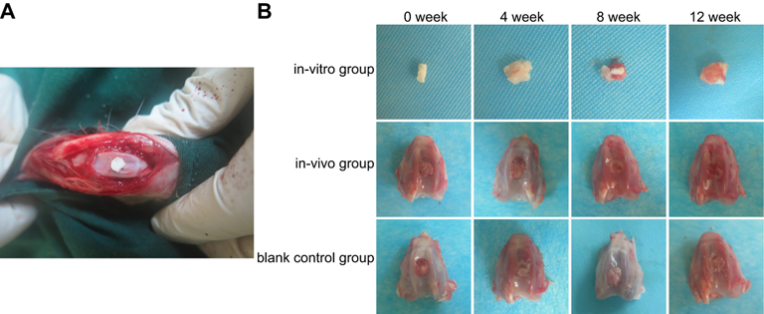
Transplantation regions, cell–scaffold compound shape and joint defect repair in each group at different time points after transplantation (**A**) Transplantation regions; (**B**) cell–scaffold compound shape and joint defect repair in each group.

DBM scaffolds composites kept original form and possessed better properties of toughness and strength, which had honeycomb-shaped and three-dimensional structure. Few parts of fibers on the surface were observed broken (Figure 3A). After composites were implanted into the knee cavities of New Zealand white rabbits, there was no observable rejection reaction. The surgical incision was dry, with no swelling or exudation, and no restriction in the movement of the knee joint was observed. No death occurred during feeding. In the *in vivo* group, the original incision healed well. When knee cavity was opened along the original incision, clear and transparent joint liquid was seen, and there was no inflammatory granulation or hyperplasia, with basically normal intra-articular environment. At the 4th week, the structure of cell–scaffold composites in the *in vitro* group had no change, but pores decreased. In the *in vivo* group, the color of repair tissues was greyish white, while the surface was smooth dense and had clear boundary with peripheral cartilages. In the blank control group, the color of repair tissues was darker, the surface was rough and also had clear boundary. After 8 weeks, the cell–scaffold composites in the *in vitro* group were soft, and parts of DBM were absorbed, while the structure collapsed slightly without significant pores on the surface. In the *in vivo* group, the color of repair tissues was similar to peripheral cartilages and the surface was rough and had clear boundary. In the blank control group, defects and concaves still existed and had clear boundary. After 12 weeks of *in vivo* culture, the cell–scaffold composites in the *in vitro* group formed into transparent cartilage-like material and their surface was rough without significant pores. In the *in vitro* group, transparent cartilage-like material was generated on the surface of the specimens with no pore inside, which was similar to the normal appearance of the cartilage. In the blank control group, surface was rough and was lower than peripheral cartilages with clear boundary (Figure 3B).

### Comparison of the chondrocytes in the *in vitro, in vivo*, and blank control groups by HE staining

**Figure 4:**
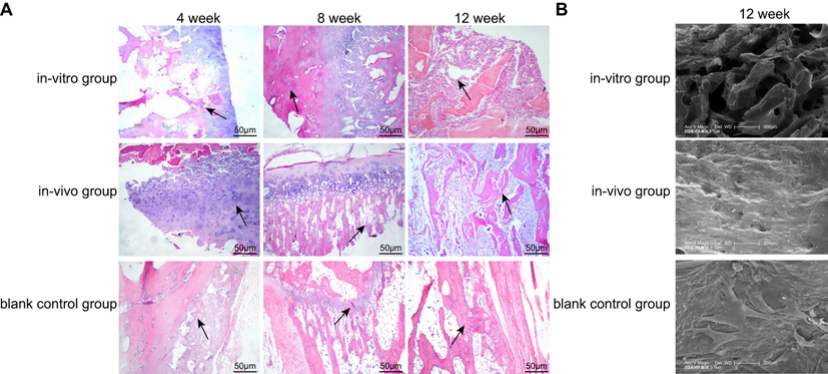
Morphology of cell–scaffold composites in different groups using HE staining at different time points and SEM at the 12th week (B) (×200) (**A**) HE staining results; (**B**) SEM results; the arrow in Figure (A) was the position of scaffold; HE, hematoxylin–eosin; SEM, scanning electron microscope.

In the 4th week, in the *in vitro* group, round or oval chondrocytes were scattered and sparsely arranged on the surface of scaffolds rather than in the interior and were surrounded by extracellular matrix. While in the *in vivo* group, chondrocytes were significantly more in the interior of scaffolds, and cartilage lacuna was formed, around which matrix was stained deeply, demonstrating that this cluster of chondrocytes was an isogenous group formed through division of a single progenitor cell. In the blank control group, DBM scaffold structure was clear, and cells analogous to chondrocytes were scattered in the interior of DBM scaffolds. Moreover, no inflammatory cell infiltration was found in any group. In the 8th week, in the *in vitro* group, the number of chondrocytes increased when compared with that in the 4th week, and chondrocytes were distributed inside the scaffold as well. While in the *in vivo* group, mature chondrocyte-based specimens had a large number of cells. The columnar arranged isogenous group of cells had a large volume, noncentrally distributed nucleus, and deeply stained nucleolus. Glassy matrix of uniform stain surrounding chondrocytes infiltrated into the interior of DBM scaffolds that were partially absorbed; and deeply stained cartilage capsule was observed around cartilage matrix. In the 12th week, scaffolds in each group were significantly absorbed, together with partial fibrosis of cancellous bone and splitting of cortical bone. In the *in vitro* group, scaffolds were filled up with chondrocytes, and fibrosis was observed in some of them in disordered arrangement. But more scaffolds in the *in vivo* group were absorbed, and chondrocytes completely infiltrated into scaffolds, and they became smaller in size and were arranged in a certain stress direction. In the blank control group, no evident absorbance was observed and the proliferation of chondrocytes was slow (Figure 4).

### A value in the *in vitro, in vivo*, and blank control groups by SP method

**Figure 5:**
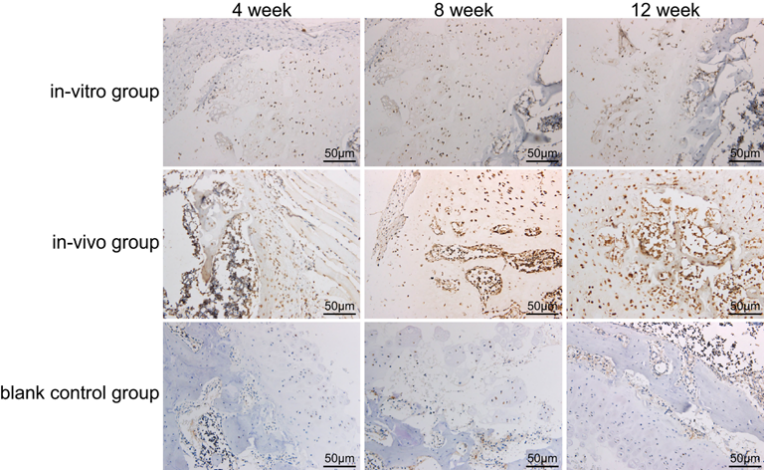
Type II collagen immunohistochemistry of all groups using SP method (×200) SP, streptavidin–peroxidase.

Type II collagen of the highest specificity generated by chondrocytes was selected as the primary antibody to perform the immunohistochemical analysis of paraffin sections. After developing, a large number of brown yellow immune complexes were found in the cytoplasm of positive cells (Figure 5). A total of eight different observations were selected from immunohistochemical sections in each group, and later fluorescence microscope and camera control system (×200) were used to take photographs, which were input into Jetta 801D morphological analysis software to calculate the *A* value. With a greater absorbance value, the light was absorbed in a greater degree, the immune complex was darker in color, and the solute content was higher. Conversely, the smaller the absorbance value, the lighter the color of the immune complex.

Compared with the *in vitro* group and the blank control group, the *A* value in the *in vivo* group increased during the 4th week (*t* = 7.696, *P*<0.01); the *A* value in the *in vivo* group increased gradually and reached the highest in the 8th week, and then began to decline, but it was still higher than that in the *in vitro* group and the blank control group (*t* = 6.301, *P*<0.01) (Table 1). The repeated measures of ANOVA were carried out and they confirmed that the data of the three groups were significantly different at the three time points (*F* = 5.130, *P*<0.05). The differences in the *A* values of the three groups were statistically significant (*P*<0.01).

**Table 1:** Comparison of the *A* value of type II collagen in each group (*n*=20, mean ± SD)

Time points	*In vitro* group	*In vivo* group	Blank control group	*t*	*P*
The 4th week	0.1043 ± 0.0290	0.2578 ± 0.0484	0.0675 ± 0.0052	7.696	<0.01
The 8th week	0.1422 ± 0.0433	0.3525 ± 0.0965	0.0886 ± 0.0064	5.622	<0.01
The 12th week	0.1480 ± 0.0381	0.3523 ± 0.0834	0.0954 ± 0.0071	6.301	<0.01

SD, standard deviation.

## Discussion

The objective of the present study was to compare two tissue-engineered cartilages cultivated from cell–scaffold composites made of allogenic DBM and cytokines-induced BMSCs both *in vivo* and *in vitro* using a rabbit knee model. The present study showed that under the hypoxic micro-environment of the knee with multiple cytokines, allogenic DBM combined with cytokine-induced BMSCs that were cultivated in knee cavity rather than *in vitro* culture generated better tissue-engineered cartilage.

Cartilage injury is a key feature of degenerative joint disorders, primarily OA and inflammatory joint diseases [15]. Due to low cell density and poor regenerative potential, cartilage has a poor ability to adequately repair itself when damaged, and the rapid development of tissue engineering is prenominal to the production of functional cartilage tissue replacement [16]. Furthermore, seed cell, scaffold material, and interaction between cytokines and culture environment are the main components for cartilage repair [17]. Although MSC is a kind of adult stem cells that is derived from the bone marrow, it is the main element for cell seeding in tissue-engineered biomaterials for bone repair [18]. Moreover, BMSCs are available in a minimally invasive manner with strong proliferation and chondrogenic potentials, which makes BMSCs an ideal cell source for tissue engineering [19]. It is also the core of cartilage tissue engineering, because DBM is an ideal scaffold material, not only for its natural porous framework of high density and better pore structure, but also for its extremely low antigenicity and good biocompatibility, which benefits the induction from bone to protein so as to facilitate the generation of bone and the cartilage [20, 21]. Besides, the culture of tissue-engineered cartilage needs cytokines to act on seed cells so as to promote the proliferation and directional differentiation, including TGF-β1, fibroblast growth factor (FGF), and IGF-1, which together regulate the differentiation of BMSCs [22]. Therefore, the chondrocytes induction solution used in the present study consisted of TGF-β1, IGF-1, and vitamin C, which provided the suitable environment for transformation of BMSCs into chondrocytes.

The present study showed that during the 8th week, in the *in vivo* group, mature chondrocyte-based specimens had a huge amount of cells and the columnar arranged isogenous group with large volume and deeply stained nucleolus, and the cells were surrounded by uniformly stained glassy matrix infiltrating in the interior of scaffold. Moreover, deeply stained cartilage capsule around the cartilage matrix was observed and parts of DBM scaffolds were absorbed as well. In the 12th week, transparent cartilage-like materials were generated on the surface of the specimens with no pore inside and they had similar appearance to the normal cartilage. One research mentioned above demonstrated that the mature chondrocyte-based specimens had abundant cells and columnar arranged isogenous group, which were surrounded by a pool of glassy matrix and were infiltrated into the interior of scaffolds, along with DBM scaffolds and were absorbed mostly and a transparent cartilage-like material was generated on the surface [23]. Besides, Qiang et al. [24] also found that in the 8th week, tissues in DBM/MSCs group were similar to the normal cartilage, and that during the 12th week, they repaired tissue in a better cell arrangement and had more cells and cartilage lacuna. Moreover their color was very similar to the surrounding cartilage. In the present study, the general observation of specimens was in accordance with the above researches, suggesting that tissue-engineered cartilage could be cultured successfully in the knee cavity of rabbit from allogenic DBM combined with rabbit BMSCs, which further certifies that the idea “culture in cavity and transplantation in cavity” is feasible and effective. In addition, the *A* value, namely average absorbance value, is directly proportional to the solute content. The present study also revealed that in the 4th week, the *A* value of type II collagen in the *in vivo* group increased, which was constantly higher than that in the other two groups, even though it dropped a little after it reached the highest point. Consistently with our results, another research demonstrated that gradually, the *A* value of the *in vivo* group was continuously increasing than that of in the *in vitro* group, indicating that the solute content was higher, which helped to get better culture effects [25].

## Conclusion

In conclusion, the present study demonstrated that allogenic DBM combined with cytokine-induced BMSCs cultivated in the knee cavity was better for the generation of tissue-engineered cartilage. It also proved that tissue-engineered cartilage generated by *in vivo* culture was better to that cultured *in vitro*. However, the present study did not compare the features between *in vivo* cultured tissue-engineered cartilage and autogenous bone, which needs further investigation in future study.

## Abbreviations

BMSC: bone marrow mesenchymal stem cell
DBM: decalcified bone matrix
DMEM: Dulbecco’s modified Eagle’s medium
HE: hematoxylin–eosin
OA: osteoarthritis
PBS: phosphate-buffered saline
SP: streptavidin–peroxidase

## References

[1] Lund S.A., Giachelli C.M. and Scatena M. (2009) The role of osteopontin in inflammatory processes. J. Cell Commun. Signal 3, 311–322 19798593 10.1007/s12079-009-0068-0 PMC2778587

[2] Wu J., Wu D., Guo K., Yuan F. and Ran B. (2014) OPN polymorphism is associated with the susceptibility to cervical spondylotic myelopathy and its outcome after anterior cervical corpectomy and fusion. Cell. Physiol. Biochem. 34, 565–574 25116355 10.1159/000363023

[3] Wang Z.C., Hou X.W., Shao J., Ji Y.J., Li L., Zhou Q. (2014) HIF-1alpha polymorphism in the susceptibility of cervical spondylotic myelopathy and its outcome after anterior cervical corpectomy and fusion treatment. PLoS One 9, e110862 25401740 10.1371/journal.pone.0110862 PMC4234507

[4] Abode-Iyamah K.O., Stoner K.E., Grossbach A.J., Viljoen S.V., McHenry C.L., Petrie M.A. (2016) Effects of brain derived neurotrophic factor Val66Met polymorphism in patients with cervical spondylotic myelopathy. J. Clin. Neurosci. 24, 117–121 26461908 10.1016/j.jocn.2015.07.016

[5] Lazebnik M., Singh M., Glatt P., Friis L.A., Berkland C.J. and Detamore M.S. (2011) Biomimetic method for combining the nucleus pulposus and annulus fibrosus for intervertebral disc tissue engineering. J. Tissue Eng. Regen. Med. 5, e179–187 21774081 10.1002/term.412

[6] Gruber H.E., Hoelscher G., Ingram J.A., Chow Y., Loeffler B. and Hanley E.N. Jr (2008) 1,25(OH)2-vitamin D3 inhibits proliferation and decreases production of monocyte chemoattractant protein-1, thrombopoietin, VEGF, and angiogenin by human annulus cells in vitro. Spine (Phila Pa 1976) 33, 755–765 18379402 10.1097/BRS.0b013e3181695d59

[7] Colombini A., Lanteri P., Lombardi G., Grasso D., Recordati C., Lovi A. (2012) Metabolic effects of vitamin D active metabolites in monolayer and micromass cultures of nucleus pulposus and annulus fibrosus cells isolated from human intervertebral disc. Int. J. Biochem. Cell Biol. 44, 1019–1030 22481027 10.1016/j.biocel.2012.03.012

[8] Li X., Jin L., Balian G., Laurencin C.T. and Greg Anderson D. (2006) Demineralized bone matrix gelatin as scaffold for osteochondral tissue engineering. Biomaterials 27, 2426–2433 16343611 10.1016/j.biomaterials.2005.11.040

[9] Holt D.J. and Grainger D.W. (2012) Demineralized bone matrix as a vehicle for delivering endogenous and exogenous therapeutics in bone repair. Adv. Drug Delivery Rev. 64, 1123–1128 10.1016/j.addr.2012.04.002 22521662

[10] Caplan A.I. (1991) Mesenchymal stem cells. J. Orthop. Res. 9, 641–650 1870029 10.1002/jor.1100090504

[11] Wang W., Li B., Yang J., Xin L., Li Y., Yin H. (2010) The restoration of full-thickness cartilage defects with BMSCs and TGF-beta 1 loaded PLGA/fibrin gel constructs. Biomaterials 31, 8964–8973 20822812 10.1016/j.biomaterials.2010.08.018

[12] Liu X.J., Chen Y., Yuan L., Yu L., Wang F. and Liao H. (2003) Morphological observation of mesenchymal stem cells cultured with allogenic decalcified bone matrix. Di Yi Jun Yi Da Xue Xue Bao 23, 40–42 12527513

[13] Chen Y., Chen Y., Zhang S., Du X. and Bai B. (2016) Parathyroid hormone-induced bone marrow mesenchymal stem cell chondrogenic differentiation and its repair of articular cartilage injury in rabbits. Med. Sci. Monit. Basic Res. 22, 132–145 27847384 10.12659/MSMBR.900242 PMC5115215

[14] Shi X.P., Zong A.N., Tao J. and Wang L.Z. (2007) Study of instructive notions with respect to caring for laboratory animals. J. China Med. Univer., 4, 493

[15] Pap T. and Korb-Pap A. (2015) Cartilage damage in osteoarthritis and rheumatoid arthritis–two unequal siblings. Nat. Rev. Rheumatol. 11, 606–615 26195338 10.1038/nrrheum.2015.95

[16] Moreira-Teixeira L.S., Georgi N., Leijten J., Wu L. and Karperien M. (2011) Cartilage tissue engineering. Endocr. Dev. 21, 102–115 21865759 10.1159/000328140

[17] Ahmed T.A. and Hincke M.T. (2010) Strategies for articular cartilage lesion repair and functional restoration. Tissue Eng. Part. B Rev. 16, 305–329 20025455 10.1089/ten.TEB.2009.0590

[18] Hou T., Li Z., Luo F., Xie Z., Wu X., Xing J. (2014) A composite demineralized bone matrix–self assembling peptide scaffold for enhancing cell and growth factor activity in bone marrow. Biomaterials 35, 5689–5699 24755526 10.1016/j.biomaterials.2014.03.079

[19] Zhang L., He A., Yin Z., Yu Z., Luo X., Liu W. (2014) Regeneration of human-ear-shaped cartilage by co-culturing human microtia chondrocytes with BMSCs. Biomaterials 35, 4878–4887 24656731 10.1016/j.biomaterials.2014.02.043

[20] Dai L., He Z., Zhang X., Hu X., Yuan L., Qiang M. (2014) One-step repair for cartilage defects in a rabbit model: a technique combining the perforated decalcified cortical-cancellous bone matrix scaffold with microfracture. Am. J. Sports Med. 42, 583–591 24496505 10.1177/0363546513518415

[21] van Bergen C.J., Kerkhoffs G.M., Ozdemir M., Korstjens C.M., Everts V., van Ruijven L.J. (2013) Demineralized bone matrix and platelet-rich plasma do not improve healing of osteochondral defects of the talus: an experimental goat study. Osteoarthritis Cartilage 21, 1746–1754 23896314 10.1016/j.joca.2013.07.014

[22] van der Kraan P.M., Buma P., van Kuppevelt T. and van den Berg W.B. (2002) Interaction of chondrocytes, extracellular matrix and growth factors: relevance for articular cartilage tissue engineering. Osteoarthritis Cartilage 10, 631–637 12479385 10.1053/joca.2002.0806

[23] Xu Bin Z.L. (2014) Chondrogenic co-culture of allogenic decalcified bone matrix and bone marrow mesenchymal stem cells in the joint cavity: comparison of cartilage traits in the same joint cavity [J]. Chin. J. Tissue Eng. Res. 18, 1165–1171

[24] Qiang L., Tang J.C. and Sun Z.Y. (2008) Repairing full-thickness articular cartilage defects with homograft of mesenchymal stem cells seeded onto cancellous demineralized bone matrix [J]. J. Clin. Rehabil. Tissue Eng. Res. 12, 8943–8947

[25] Hare J.M., Fishman J.E., Gerstenblith G., DiFede Velazquez D.L., Zambrano J.P., Suncion V.Y. (2012) Comparison of allogeneic vs autologous bone marrow-derived mesenchymal stem cells delivered by transendocardial injection in patients with ischemic cardiomyopathy: the POSEIDON randomized trial. J. Am. Med. Assoc. 308, 2369–2379 10.1001/jama.2012.25321 PMC4762261 23117550

